# A novel model of adenine-induced tubulointerstitial nephropathy in mice

**DOI:** 10.1186/1471-2369-14-116

**Published:** 2013-05-30

**Authors:** Ting Jia, Hannes Olauson, Karolina Lindberg, Risul Amin, Karin Edvardsson, Bengt Lindholm, Göran Andersson, Annika Wernerson, Yves Sabbagh, Susan Schiavi, Tobias E Larsson

**Affiliations:** 1 Department of Clinical Science, Intervention and Technology, Karolinska Institutet, Stockholm, Sweden; 2 Renal Medicine and Baxter Novum, Karolinska Institutet, Stockholm, Sweden; 3 Department of Pathology, Karolinska Institutet, Stockholm, Sweden; 4 Sanofi-Genzyme R&D Center, Genzyme, A Sanofi Company, Framingham, USA; 5 Department of Nephrology, Karolinska University Hospital, Stockholm, Sweden

**Keywords:** CKD, Chronic kidney disease, CKD-MBD, FGF-23, FGF23, Mineral metabolism, Experimental renal failure

## Abstract

**Background:**

*In vivo* models of uremia are important tools to study numerous aspects of acute and chronic kidney disease. Mouse models are pivotal because most genetically engineered animal models are mice, which allow dissecting the impact of selected target genes in renal failure. Adenine-based protocols to induce renal failure are available in rats, but have not been adapted in mice due to their reluctance to consume adenine. In the current paper we developed a novel method for induction of renal failure through dietary delivery of adenine mixed in a casein-based diet.

**Results:**

After an induction phase, a stable model of renal impairment was obtained (target urea range 80–100 mg/dL), mimicking several aspects of chronic kidney disease - mineral and bone disorder including secondary hyperparathyroidism, bone abnormalities and pathological elevation of FGF23. No deaths occurred and the level of uremia was adaptable through adjustments of the adenine content, providing significant advantages compared to existing models. In an 8-week proof-of-concept study, renal histology showed mainly a tubulointerstitial damage with infiltrating leukocytes, interstitial edema and widening of the Bownman's space. Fibrosis was present in most animals as defined by histology and gene expression changes of fibrosis markers. Parathyroid cell proliferation was markedly increased but without signs of glandular hypertrophy. Skeletal histology showed increased trabecular bone and bone marrow adiposity whereas bone biomarkers (CTX and PINP) suggested higher bone formation, but surprisingly, lower bone resorption and perturbations in mineral metabolism.

**Conclusions:**

We present a novel, non-surgical method for induction of renal failure in mice. This is an important complement to existing uremic models for pathophysiological studies in acute and chronic kidney disease, especially in terms of tubulointerstitial lesions.

## Background

Chronic kidney disease (CKD) is a global health burden [1], yet effective treatments for its prevention, progression and associated complications are currently lacking. Animal models of renal failure are important tools to study pathophysiological events in kidney disease that allows translational studies aiming to improve management of CKD patients. Due to the high availability of genetically engineered mouse strains, uremic mouse models provide the opportunity to investigate the impact of specific target genes in the setting of renal failure.

Established techniques for induction of renal failure in mice are mostly dependent on surgical interventions. The most widespread methods currently used are unilateral ureteral obstruction [2-7], which leads to interstitial fibrosis by infiltration of macrophages and tubular cell death by apoptosis and necrosis, and 5/6 nephrectomy [8,9]. The latter technique usually involves two separate procedures, first two thirds of one kidney is destructed by electrocoagulation and after recovery a contra-lateral nephrectomy is performed. The 5/6 nephrectomy model has several limitations including a substantial mortality rate when not performed adequately, non-reversibility and phenotypic alterations related to the surgical procedure rather than impaired kidney function [10]. The method is also associated with relatively large inter-individual and inter-laboratory variations and its availability may be compromised by lack of surgical expertise and the appropriate operating facilities.

To circumvent these obstacles, we aimed at establishing a novel, non-surgical model of renal failure in mice by employing an adenine-based protocol. Importantly, there are well-characterized protocols for adenine-induced renal failure in rats, yet this technique has not been adapted in mice due to their aversion to adenine feeding.

## Methods

### Animal experiments

The experiments were conducted in compliance with the guidelines of animal experiments of Karolinska Institutet and the study protocol was approved by the regional ethical committee (Stockholm South ethical committee, approval number S184-10 and appendix S19-13). 8-week-old C57BL/6J mice were housed in standard cages with wood chip bedding and a paper roll for enrichment at constant ambient temperature (21–22°C) and humidity (40-50%) with a 12-hour light cycle. All animals had free access to tap water and the assigned diet. Before study start, all mice were allowed acclimatization to the animal facility conditions and the casein-based chow during a 7-day period.

To provide an adenine-containing chow consumed by the mice, adenine was mixed with a casein-based diet that blunted the smell and taste. Adenine was purchased from Sigma Aldrich (MO, USA) and the powdered casein-based diet from Special Diets Services (SDS, UK) (reference number 824522). Other ingredients of the diet are maize starch (39.3%), casein (20.0%), maltodextrin (14.0%), sucrose (9.2%), maize/corn oil (5%), cellulose (5%), vitamin mix (1.0%), DL-methionine (0.3%) and choline bitartrate (0.2%). Total phosphate content was 0.9% and total calcium content was 0.6%.

Data presented herein is derived from an 8-week experiment using 8-week-old C57BL/6J wild-type mice. To exclude the possibility of an impact of the casein diet per see on renal function, the control group (n = 5) was fed the same casein diet as the adenine group (n = 9) but without addition of adenine.

### Serum and urine biochemistries

Blood was collected after cardiac puncture at sacrifice and by tail vein incision at intermediate time points. Urine was collected as spot urine samples after spontaneous urination. Serum and urine calcium, phosphorous, creatinine and urea levels were measured on a Konelab 20XTi (Thermo Scientific, Finland). Creatinine concentrations were validated with a colorimetric assay (BioChain, CA, USA) yielding nearly identical results (rho = 0.95 and 0.98 for serum and urine creatinine respectively) when compared with the Konelab technique. PTH was measured by a mouse intact PTH ELISA kit (Immutopics, CA, USA), FGF23 with an intact FGF23 ELISA (Kainos, Japan) and 1,25(OH)_2_D, CTX and PINP with EIA kits (Immunodiagnostic Systems, UK).

### Histology

Kidneys and parathyroid glands were fixed in 4% formaldehyde, embedded in paraffin and sectioned according to standard procedures. Bones were decalcified in a buffer containing 20% formic acid. All tissues were subject to Hematoxylin and Eosin staining. Kidney sections were also stained for Periodic acid-Schiff stain (PAS) for polysaccharides and mucosubstances (VWR International, Sweden), Trichrome (Ladewig) for muscle and collagen (Histolab Products AB, Sweden) and von Kossa for mineralized tissue (VWR International), according to the manufacturers instructions. The kidneys and bones were evaluated in a blinded fashion by an experienced kidney pathologist (AW) and bone pathologist (GA), respectively. Immunohistochemistry was performed according to standard protocols using a rabbit monoclonal anti-Ki67 antibody (SP6 1:400, Thermo Scientific, CA, US) and a rabbit anti-human Myeloperoxidase antibody (A398 1:100, Dako, Denmark). Proliferation index in parathyroid glands was calculated as the number of Ki67 positive cells divided by total number of cells in four consecutive sections.

### Alizarin red and vascular calcification

The thoracic to abdominal part of the aorta (n ≥ 5 from each group) was dissected, split longitudinally and laid flat. The tissue was incubated with 1% alizarin red S-staining solution (Alfa Aesar, Germany) at room temperature for 10 minutes and washed three times with 70% ethanol for 20 minutes each.

### Analysis of renal transcripts

Kidneys were homogenized using a TissueLyzer LT (Qiagen, Netherlands) and total RNA was extracted using E.Z.N.A. Total RNA Kit I (Omega Bio-tek, GA, US). DNA was removed with E.Z.N.A. RNase-Free DNase Set (Omega Bio-tek). First-strand cDNA was synthesized using iScript cDNA Synthesis Kit (Bio-Rad, Hercules, CA, US).

For real time qPCR analysis the CFX96 Real-Time PCR Detection System and iQ SYBR Green Supermix (Bio-Rad) were used. The relative gene expression was calculated using the 2^-ΔΔ^ Cq method normalizing the gene of interest to β-actin in the same sample.

### Statistics

GraphPad Prism 5.0 (GraphPad Software Inc, CA, US) was used for statistical analysis. All values are expressed as mean ± SEM unless otherwise stated. Differences between adenine-treated mice and controls were calculated using the Mann–Whitney non-parametric test. *P*-values <0.05 were considered statistically significant.

## Results

### Protocol development

We performed a series of pilot studies to establish the optimal degree of renal impairment at which common biochemical abnormalities associated with renal dysfunction occurred without increased morbidity or mortality. For this purpose, serum urea levels ranging from 80–100 mg/dL were considered optimal. Urea levels above 100 mg/dL were associated with increased morbidity (weight loss and reduced physical activity), whereas urea concentrations of <70-80 mg/dL led to a weight gain and a partial reversal of biomarkers associated with impaired kidney function such as phosphorous and PTH. Importantly, the casein-diet without addition of adenine was used as control diet and did not influence serum urea and creatinine levels or renal histology compared to mice on a regular diet (data not shown).

The required adenine dose to achieve the target urea interval was determined to 0.3% adenine during a 10-day “induction phase” and 0.15-0.20% during a “maintenance phase”. When adhering to this urea range, no deaths occurred during the entire study or in supporting pilot studies. Accordingly, we propose that the adenine concentration can be modified *ad libitum* between 0.15-0.20% during the maintenance phase. Notably, we also evaluated adenine delivery by gavage, which resulted in an approximately 30% overall mortality and higher variability in urea level and is therefore not recommended.

### Proof-of-concept study

**Figure 1:**
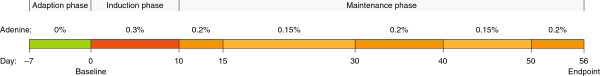
**Schematic view of the 8-week proof-of-concept study of adenine-induced renal failure in mice.** The study was preceded by a 7-day adaptation phase, and comprised a 10-day induction phase (day 0–9) and a maintenance phase (day 10–56).

We performed an 8-week experiment, a time frame generally endorsed in other uremic models of renal failure to study CKD complications such as vascular calcification [10]. The study protocol is shown in Figure 1. The study was preceded by a 7-day adaptation phase of the casein diet without addition of adenine, and comprised a 10-day induction phase (day 0–9) and a maintenance phase (day 10–56). During the maintenance phase, the adenine content was modified as described above in the interval 0.15-0.20% to achieve the desired urea level of 80–100 mg/dL. Of note, lowering the adenine concentration on day 15 and 40, from 0.2% to 0.15%, resulted in a rapid decline in urea and PTH levels. However, long-term reversibility and histological improvements after adenine withdrawal was not examined.

### Body weight and serum/urine biochemistries

**Figure 2:**
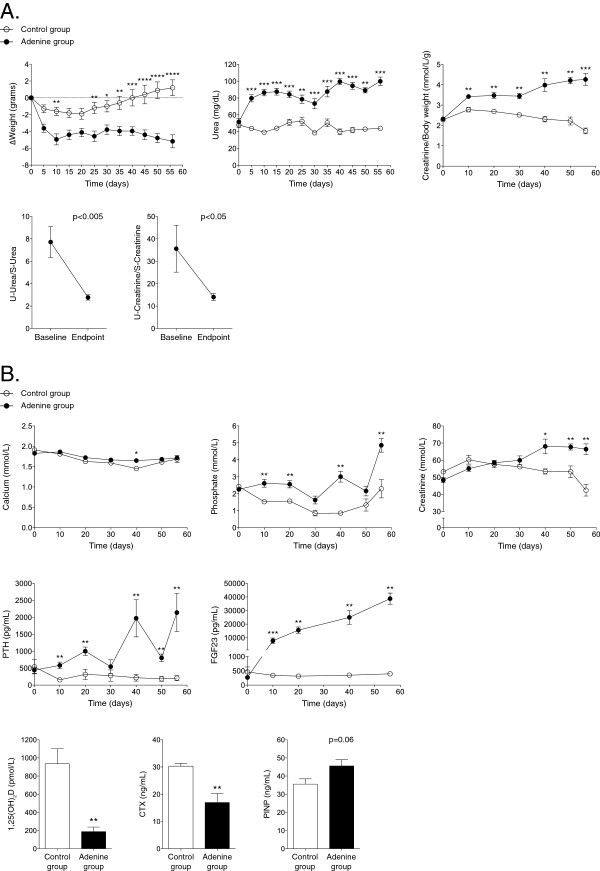
**Body weight and biochemical parameters during the study. A**: Body weight and renal function parameters during the study. ΔBody weight (top): Body weight was reduced during the 10-day induction phase in the adenine-treated group but remained stable during the maintenance phase until endpoint. *Markers of kidney function:* serum urea, serum creatinine/body weight, urine urea/serum urea and urine creatinine/serum creatinine indicated reduced renal clearance rate in adenine-treated mice. *p < 0.05; **p < 0.01; ***p < 0.001; ****p < 0.0001. **B**: Temporal changes in serum biochemistries of mineral metabolism. At endpoint, there was a significant increase in serum inorganic phosphorous, PTH and FGF23 but a decrease in 1,25(OH)_2_D level in adenine-treated mice. The bone marker CTX was decreased and PINP borderline increased in the adenine group, supporting a reduced bone resorption but increased bone formation. *p < 0.05; **p < 0.01.

The temporal changes in body weight and markers of kidney function are depicted in Figure 2A. There was a significant decline in body weight during the induction phase but it was stabilized and essentially unaltered during the maintenance phase. In accordance with previous reports urea appeared to be a more accurate marker of uremia than creatinine, whereas the ratio of creatinine/body weight paralleled the urea levels [11]. Decreased ratios between urine urea/serum urea and urine creatinine/serum creatinine confirmed reduced clearance of these metabolites (Figure 2A). Importantly, the adenine-exposed mice did not have increased proteinuria compared to controls. This is likely explained by the C57BL/6 strain's known resistance towards development of proteinuria in combination with the tubulointerstitial nature of the renal damage in this model [12].

Markers of bone and mineral metabolism are shown in Figure 2B. Similar to CKD patients, the adenine group developed a significant hyperphosphatemia, secondary hyperparathyroidism and severely elevated FGF23 paralleled by an increased urinary excretion of phosphorous [13]. There was no change in urinary excretion of calcium (p = 0.66 for baseline versus endpoint). At endpoint, CTX was significantly decreased whereas there was a borderline significant increase in PINP in adenine-treated mice compared to mice on a control diet, suggesting a reduced bone resorption but an increased bone formation (Figure 2B).

### Histology

**Figure 3:**
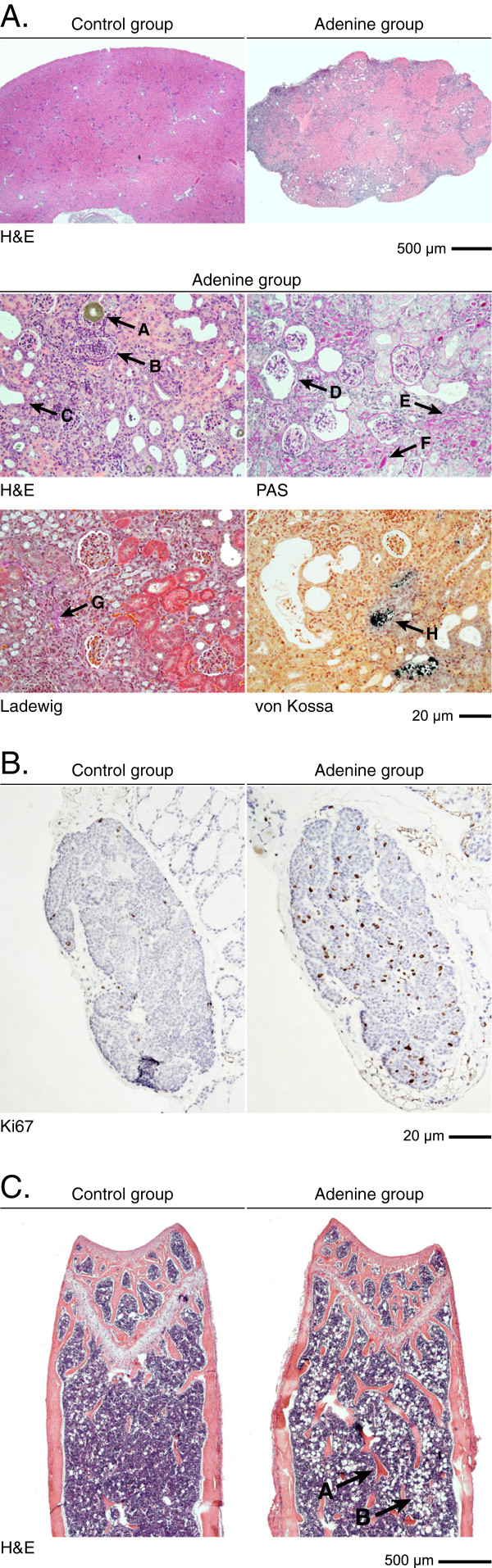
**Histological analyses of kidneys, parathyroid glands and bones. A**: Renal histology showed deposition of symmetric crystalline structures in a tubular lumen (arrow A), micro abscesses (B) and dilated tubules (C). PAS staining showed dilated Bowman’s space (D), atrophic tubuli with protein casts (“thyroidization”) (E) and tubular atrophy with thickening of the tubular basement membrane (F). Ladewig staining revealed a mild interstitial fibrosis (G). Extensive calcification of tubular structures (H) was seen with von Kossa staining. **B**: Parathyroid glands were not hypertrophic but had a significantly increased proliferation rate determined by Ki67 index (8.7 ± 0.7% vs 2.3 ± 0.5%; *p* < 0.0001). **C**: In bone, there was an increased number and thickness of submetaphyseal bone trabeculae (arrow A) and increased adipocyte content in the bone marrow (B).

Histological analysis of kidneys, parathyroid glands and bone are shown in Figure 3A-C and in Additional file 1: Figure S1. Renal histology showed a peritubular leukocyte infiltration and interstitial/peritubular edema reflecting that the kidney damage is mainly tubulointerstitial. Positive immunostaining for Myeloperoxidase confirmed that the peritubular leukocytes were mainly comprised of neutrophil granulocytes. (Additional file 2: Figure S2). An increase in the Bowman´s space was seen in some but not all glomeruli. Focal micro-abscesses were present in some, but not all, tissue specimens examined in the adenine-group. A summary of histopathological findings in the kidney is provided in Table 1.

**Table 1:** Histopathological evaluation of kidneys from control and adenine-treated mice

**Compartment**	**Parameter**	**Control group**	**Adenine group**
		**(n = 5)**	**(n = 8)**
**Glomeruli**	Sclerosis (present/absent)	0/5	0/8
	Dilatation of Bowmans capsules (present/absent)	0/5	5/3
**Tubuli**	Rounded cristalloid/amorphous structures in tubular lumina (present/absent)	0/5	8/0
	Cell debris/necrotic material and PMN in tubular lumina (0–3)	0 (0–0)	1 (1–1)
	Tubular atrophy (0–3)	0 (0–0)	2 (2–3)
	Thyroidization (0–3)	0 (0–0)	1 (1–2)
	Focal dilatation of tubuli (present/absent)	0/5	6/2
	Focal calcium deposits (present/absent)	0/5	5/3
**Interstitium**	Fibrosis (0–3)	0 (0–0)	1 (1–1)
	Inflammation (0–3)	0 (0–0)	0 (0–1)
**Vessels**	Morphology (pathological/normal)	0/5	0/8

The parameters tubular atrophy, cell debris/necrotic material and polymorphonuclear leukocytes (PMN) in tubular lumina, thyroidization, interstitial fibrosis and interstitial inflammation are graded as follows: 0; affecting 0-5% of the renal area, 1; 6-25%, 2; 26-50%, and 3; >50%. Data is presented as median (range). Rounded cristalloid/amorphous structures in tubular lumina, focal dilatation and focal calcium deposits in tubuli are graded as present or absent. Vessel morphology is graded as pathological or normal.

Areal determination from serial sectioned parathyroid glands revealed no overt glandular hypertrophy. Conversely, proliferation rate in the parathyroids determined by Ki67 index was increased (Figure 3B), and in line with the increased PTH level in adenine-treated mice. Sections of femurs showed an extended bone trabeculae and increased bone marrow adiposity consistent with the serum pattern of the bone markers PINP and CTX. Alizarin red stainings did not show any overt vascular calcification in the thoracic aortas of adenine-exposed mice (Additional file 3: Figure S3).

### Gene expression changes of local inflammatory and fibrosis markers

**Figure 4:**
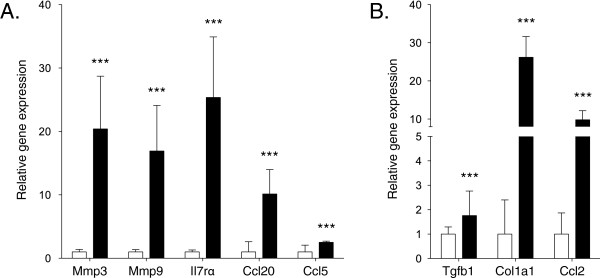
**Expression of renal-derived inflammatory and fibrosis genes. A**) Inflammatory markers Mmp3, Mmp9, Il7rα, Ccl20 and Ccl5, and **B**) Marker of fibrosis Tgfb1, Col1a1 and Ccl2 were upregulated in adenine treated mice. Relative gene expression in the control group is set to 1. White bars; control group, black bars; adenine group. ***p < 0.001.

We analyzed the transcript levels of a number of locally activated genes associated to renal inflammation and fibrosis [14,15]. Inflammatory markers Mmp3 (Gene ID: 17392), Mmp9 (17395), Il7rα (16197), Ccl20 (20297) and Ccl5 (20304) were all significantly upregulated in the adenine-treated mice (Figure 4A), whereas Cxcr2 was unchanged (data not shown). Corresponding markers of fibrosis Tgfb1 (21803), Col1a1 (12842) and Ccl2 (20296) were significantly upregulated in mice on an adenine diet (Figure 4B). Primers used for real time qPCR analysis are presented in Additional file 4: Table S1.

## Discussion

Mouse models of uremia are important tools for *in vivo* translational studies such as examining the impact of specific genes in transgenic or knockout mice and to validate potential therapeutic interventions prior to pre-clinical testing in humans. The limitations of existing surgical models of uremia in mice, including requirements of surgical skills, demand of animal facilities to support post-operative care, high mortality rate and less flexibility in terms of dynamic alterations in urea levels, prompted us to develop a non-surgical model of renal dysfunction based on dietary intake of adenine. This approach has successfully been used in rats but not in mice because of their reluctance to consume adenine. Accordingly, addition of adenine to a standard mouse chow elicits high morbidity and mortality due to starvation and malnutrition rather than renal failure. In our model, this was circumvented by mixing the adenine in a casein-based chow, in which the casein effectively removed the inherent smell and taste of adenine.

Since the sensitivity to adenine as well as food intake may vary between various strains of mice, we propose a protocol with *ad libitum* changes in adenine concentration between 0.15-0.20% during the maintenance phase. The accepted variability in blood urea level during the maintenance phase may cause inter-individual variations in kidney function that could affect the phenotype. However such variability is inevitable and present in other uremic animal models as well. On the contrary, providing a target urea interval provides the opportunity to adjust blood urea levels according to the desired outcome or mechanisms of interest.

The apparent improvement in kidney function after lowering the adenine concentration, as indicated by a decline in blood urea and PTH levels, suggests at least a partial reversibility of renal impairment. However we did not examine whether adenine discontinuation translated into long-term histological improvements. Given the severity of the renal histopathological lesions observed after eight weeks of adenine exposure, we anticipate that long-term adenine challenge will cause chronic renal failure with less reversibility as observed in adenine-induced uremia in rats [16]. Regardless, the possibility to short-term modulate kidney function provides significant benefits in terms of the possibility to extend study protocols and to analyze outcome variables in various strata of kidney function. Additional advantages of our model include zero mortality, which limits the number of animals needed for induction, and the small inter-individual variation in renal function that contrasts the 5/6 nephrectomy model [10,17]. It also provides a good option for researchers with limited surgical competence and/or restrictions in post-operative care.

Some more common non-surgical options to study uremia including radiation nephropathy and administration of nephrotoxic drugs such as folic acid [18], cyclosporin A [19] and cisplatin [20] should be mentioned. However, these models are non-reversible, strain-dependent and of limited use due to systemic toxicity. Genetic mouse models mimicking various aspects of kidney failure are also available, but these are compromised by the need for breeding to create combined backgrounds with other genetically altered mouse strains [20].

Several different uremic models have also been developed in rats. Advantages with rat models are that collected blood and urine volumes are significantly greater, blood samples at intermediate time points are more easily obtainable and certain organs such as parathyroid glands are readily identified. Another apparent difference is that rats tolerate higher adenine exposure, which reportedly produce 3–5 times higher blood urea concentrations than our model. This may also impact renal histology since rats in additional to tubular damage generally also suffer from extensive glomerular damage which was not found in our model [21].

There are several biochemical findings of interest in our model. Serum calcium level was unaltered in the adenine-treated mice likely due to a compensatory rise in PTH, which largely mimics the situation in patients with CKD stage 3–4. Normal serum calcium concentrations have also been reported in other CKD models [22]. Another striking finding is the continuous and marked rise in FGF23 although other bone-mineral markers remained more constant during the maintenance phase. This supports the presence of renal derived factors that regulates FGF23 synthesis in bone. Alternatively, the tubular damage may severely hamper intact FGF23 degradation leading to accumulation of circulating FGF23 protein. The pattern of bone markers suggesting an increased bone formation but decreased bone resorption is somewhat unexpected in a uremic model of secondary hyperparathyroidism and anticipated high bone turnover rate. The mechanism(s) underlying reduced bone resorption are unclear but could speculatively be due to impaired osteoclast function as a result of the exceptionally elevated levels of FGF23.

Some potential limitations should be mentioned. We cannot exclude the possibility of systemic toxicity or organ-specific damages caused by the adenine. Because tubular toxicity of adenine metabolites is the underlying mechanism of adenine-induced renal failure [23,24], our model primary reflects a tubulointerstitial disease whereas the most common cause of CKD in human is glomerular scarring secondary to vascular damage. Thus, our model should not be regarded as a model of CKD *per se* but rather as a complementary model of renal failure. We did not determine the dietary (caloric) intake, yet based on previous experiments in rats the adenine-fed animals may have somewhat lower overall dietary intake. Further, we did not perform dynamic bone histomorphometry although adenine models in rats produce a high turnover phenotype. Finally, possible strain differences as found in other mouse models [25] warrant further investigation.

## Conclusions

We present a novel non-surgical protocol for induction of renal failure in mice. This will be an important complement to existing models for the study of pathophysiological events and complications related to acute and chronic kidney disease, specifically in terms of tubulointerstitial lesions and abnormalities in mineral metabolism.

## Abbreviations

CKD: Chronic kidney disease; FGF23: Fibroblast growth factor-23; EIA: Enzyme-linked immunoassay; PTH: Parathyroid hormone; CTX: Carboxyl-terminal collagen crosslinks; PINP: Procollagen type I N-terminus propeptide.

## Competing interests

The authors declare that they have no competing interests.

## Authors’ contributions

TJ: Acquisition and analysis of data; drafting and revision of manuscript; statistical analysis. HO: Acquisition and analysis of data; drafting and revision of manuscript; statistical analysis. KL: Data collection; revision of manuscript. RA: Data collection; revision of manuscript. KE: Data collection; revision of manuscript. BL: Revision of manuscript. GA: Acquisition and analysis of bone data; revision of manuscript. AW: Acquisition and analysis of renal histology; revision of manuscript. YS: Conception and design of study; revision of manuscript. SS: Conception and design of study; revision of manuscript. TEL: Conception and design of study; data analysis; drafting and revision of manuscript. All authors read and approved the final manuscript.

## Pre-publication history

The pre-publication history for this paper can be accessed here:

http://www.biomedcentral.com/1471-2369/14/116/prepub

## Supplementary Material

Additional file 1: Figure S1 High resolution images of renal histology. Hematoxylin and eosin stain (upper left panel) showed deposition of symmetric crystalline structures in a tubular lumen, micro abscesses and dilated tubules. PAS stain (upper right panel) showed dilated Bowman’s space, atrophic tubuli with protein casts (“thyroidization”) and tubular atrophy with thickening of the tubular basement membrane. Ladewig stain (lower left panel) revealed a mild interstitial fibrosis. Extensive calcification of tubular structures was seen with von Kossa stain (lower right panel). Click here for file

Additional file 2: Figure S2 Positive immunostaining for Myeloperoxidase confirmed that the peritubular leukocytes were mainly comprised of neutrophil granulocytes. Click here for file

Additional file 3: Figure S3 Alizarin red S-staining of representative segments from thoracic aorta. No vascular calcification was found in control or adenine-treated mice. Click here for file

Additional file 4: Table S1 List of primers used for real time qPCR analysis. Click here for file
