# Supplementary figures and images for: A novel model of adenine-induced tubulointerstitial nephropathy in mice
Source: BMC Nephrol. 2013 May 30;14:116. doi: 10.1186/1471-2369-14-116 (PMC3682934; doi:10.1186/1471-2369-14-116)

Supplemental Figure 1

H&E

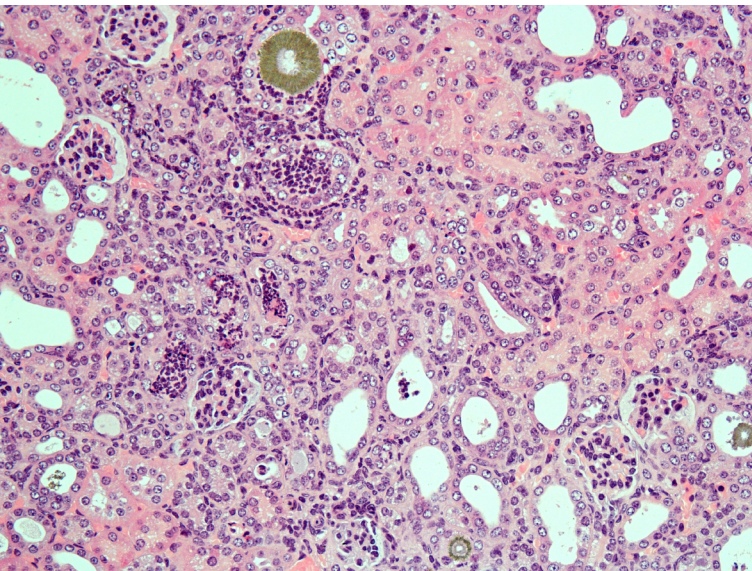

PAS

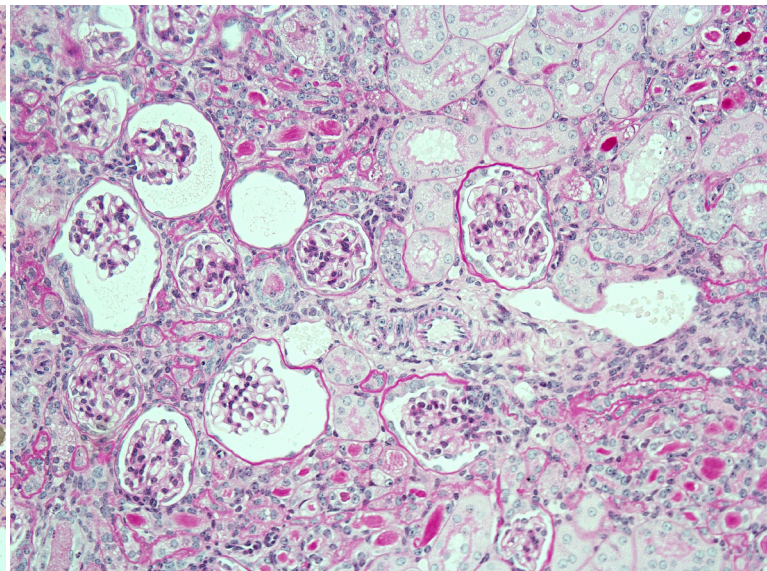

Ladewig

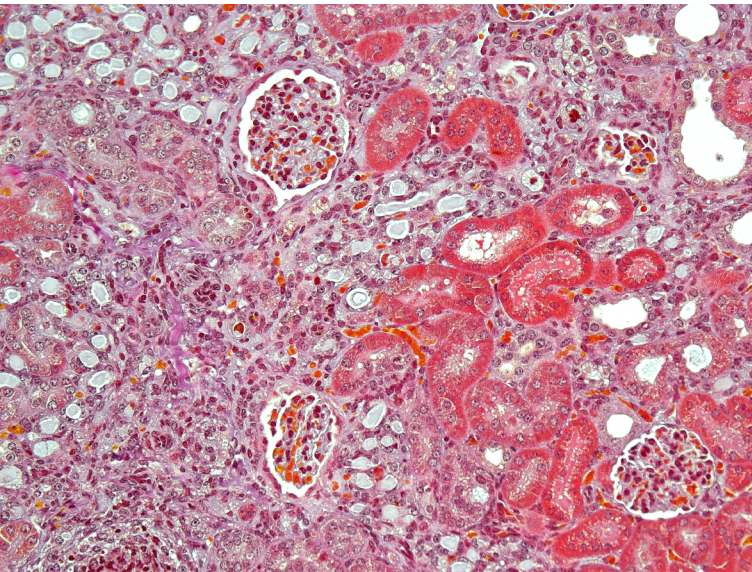

von Kossa

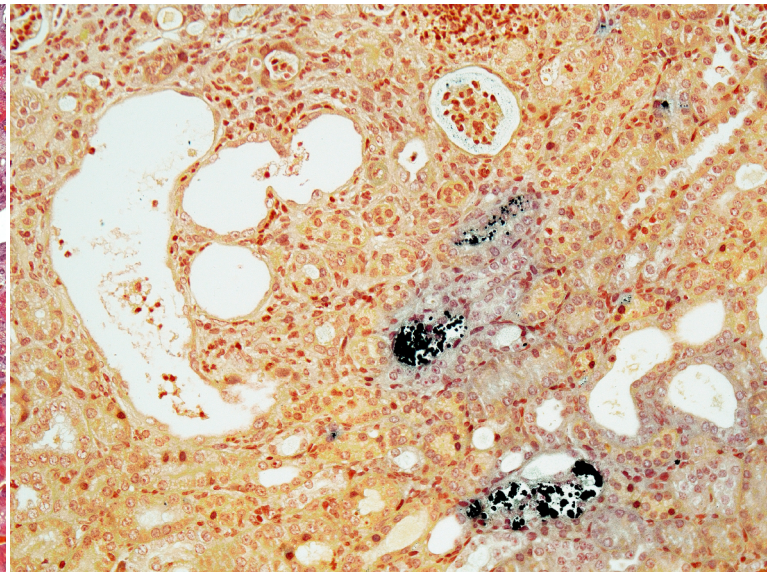

Supplement: Additional file 1: Figure S1 — High resolution images of renal histology. Hematoxylin and eosin stain (upper left panel) showed deposition of symmetric crystalline structures in a tubular lumen, micro abscesses and dilated tubules. PAS stain (upper right panel) showed dilated Bowman’s space, atrophic tubuli with protein casts (“thyroidization”) and tubular atrophy with thickening of the tubular basement membrane. Ladewig stain (lower left panel) revealed a mild interstitial fibrosis. Extensive calcification of tubular structures was seen with von Kossa stain (lower right panel). [file 1471-2369-14-116-S1.pdf]

## Supplemental Figure 2

### Myeloperoxidase

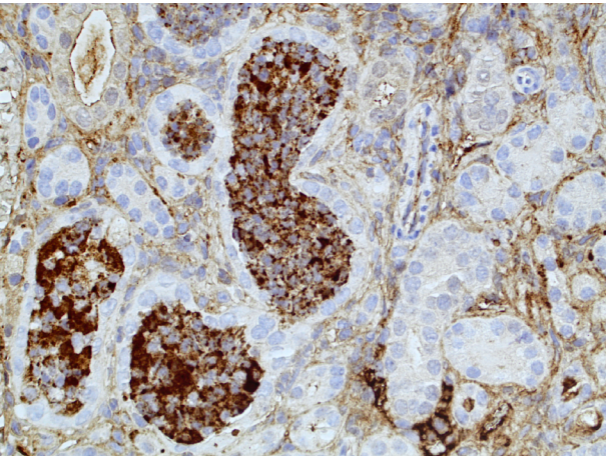

Supplement: Additional file 2: Figure S2 — Positive immunostaining for Myeloperoxidase confirmed that the peritubular leukocytes were mainly comprised of neutrophil granulocytes. [file 1471-2369-14-116-S2.pdf]

# Supplemental Figure 3

Control group

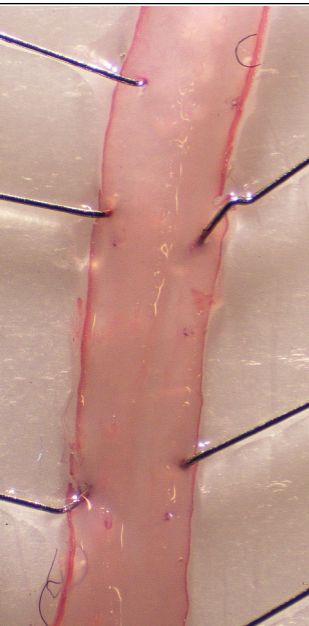

Adenine group

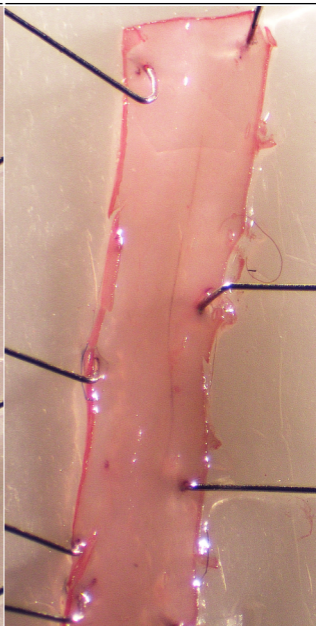

Supplement: Additional file 3: Figure S3 — Alizarin red S-staining of representative segments from thoracic aorta. No vascular calcification was found in control or adenine-treated mice. [file 1471-2369-14-116-S3.pdf]
