# Supplementary material for: A novel model of adenine-induced tubulointerstitial nephropathy in mice
Source: BMC Nephrol. 2013 May 30;14:116. doi: 10.1186/1471-2369-14-116 (PMC3682934; doi:10.1186/1471-2369-14-116)
Supplement: Additional file 4: Table S1 — List of primers used for real time qPCR analysis. [file 1471-2369-14-116-S4.doc]

**Supplemental Table 1**

| **Target gene**  **(real time qPCR)** | **Forward** | **Reverse** |
| --- | --- | --- |
| β-actin | CCG TAA AGA CCT CTA TGC CAA CAC | GAG CCA CCG ATC CAC ACA GA |
| Ccl2 (MCP-1) | CTT CTG GGC CTG CTG TTC A | CCA GCC TAC TCA TTG GGA TCA |
| Ccl5 | CAA GTG CTC CAA TCT TGC AGT C | TTC TCT GGG TTG GCA CAC AC |
| Ccl20 | TGG GTA CTG CTG GCT CAC CT | CGA GAG GCA ACA GTC GTA GTT G |
| Col1a | TGC CGT GAC CTC AAG ATG TG | CAC AAG CGT GCT GTA GGT GA |
| Cxcr2 | ATG CCC TCT ATT CTG CCA GAT | GTG CTC CGG TTG TAT AAG ATG AC |
| Il7rα | CGA AAC TCC AGA ACC CAA GA | AAT GGT GAC ACT TGG CAA GAC |
| Mmp3 | ACA TGG AGA CTT TGT CCC TTT TG | TTG GCT GAG TGG TAG AGT CCC |
| Mmp9 | CAT CCA GTA TCT GTA TGG TCG TG | GCT GTG GTT CAG TTG TGG TG |
| Tgfb1 | CAA CAA TTC CTG GCG TTA CCT TGG | GAA AGC CCT GTA TTC CGT CTC CTT |
